# Epidemiology and comprehensive economic impact of atrial fibrillation and associated stroke in Slovakia

**DOI:** 10.1186/s12913-024-11100-1

**Published:** 2024-05-17

**Authors:** Robert Babela, Anna Baráková, Robert Hatala

**Affiliations:** 1 Slovak Medical University, Bratislava, Slovakia; 2 PHC, Project HealthCare, Bratislava, Slovakia; 3 Slovak Heart Rhythm Association (SASA), Bratislava, Slovakia; 4 National Institute of Cardiovascular Diseases, Bratislava, Slovakia

**Keywords:** Atrial fibrillation, Stroke, Cost of illness, Health care, Productivity loss

## Abstract

**Background:**

Atrial fibrillation (AF) is the most common sustained cardiac arrhythmia. It is also a major risk factor for ischemic stroke. The main objective of our study was to identify direct and indirect costs of AF and AF-related stroke in Slovakia.

**Methods:**

We conducted a retrospective population-based study of AF and stroke related costs both from the third-party healthcare payers and societal perspective. The prevalence and incidence of AF and stroke were determined from central government run healthcare database. Further we estimated both indirect and direct costs of AF and stroke. All costs and healthcare resources were assessed from 2015 through 2019 and were expressed in the respective year.

**Results:**

Over the 5-year study period, the prevalence of AF increased by 26% to a total of 149,198 AF cases in 2019, with an estimated total annual economic burden of €66,242,359. Direct medical costs accounted for 94% of the total cost of AF. The total cost of treating patients with stroke in 2019 was estimated at €89,505,669. As a result, the medical costs of stroke that develops as a complication of AF have been estimated to be €25,734,080 in 2019.

**Conclusions:**

Our study shows a substantial economic burden of AF and AF-related stroke in Slovakia. In view of the above, both screening for asymptomatic AF in high-risk populations and effective early management of AF with a focused on thromboprophylaxis rhythm control should be implemented.

**Supplementary Information:**

The online version contains supplementary material available at 10.1186/s12913-024-11100-1.

## Background

Atrial fibrillation (AF) is the most prevalent cardiac arrhythmia in the adult Western population. Recent epidemiological studies using long-term electrocardiographic monitoring have revealed that the overall prevalence of AF in the general adult population of Europe varies from 1.9% in Italy to even 3.0% in Sweden [1]. Albeit AF is asymptomatic in a significant proportion of patients (up to 70%), its major public health threat is the associated high risk of thromboembolic stroke. Presence of AF increases the risk of stroke fivefold compared to sinus rhythm [2]. Thromboembolic stroke due to AF are usually large in extent and cause early death or significant permanent brain damage with devastating impact on patient independence. However, appropriate thromboprophylaxis using anticoagulants reduces the risk of stroke by 64% [3].

Data from Slovakia are relatively scarce and were acquired by various approximative methodologies. In an international survey it was estimated that in 2019 1.95% of the Slovak population suffered from AF, and a similar percentage from stroke, which was—surprisingly—slightly below the average prevalence in other Central European countries. The epidemiological landscape of AF and stroke in Slovakia remained consistent over the last 10 years in terms of incidence rates (132 per 100,000 population in 2010, and 126/100,000 in 2019 for AF; 224/100,000 in 2010, and 226/100,000 in 2019 for stroke) [4]. Similarly, no change was observed for the rate of death due to stroke, which was estimated at 108 per 100,000 population in both 2010 and 2019, positioning itself below the average for the Central European countries (187/100,000 in 2019) [5]. Stroke accounted for 10.8% of deaths from all causes and for 6.54% of disability-adjusted life years from all causes in 2019 in Slovakia [6]. Projections for the impact of stroke in Slovakia showed a dramatic increase in the number of prevalent cases from 139,000 to 201,500 by the year 2047 [7]. With the current and anticipated magnitude of clinical burden of AF and stroke in Slovak population, an associated economic impact on direct and indirect costs can be expected.

To our best knowledge, there are no published data on the economic impact of AF and AF-related stroke from both healthcare payer and societal perspective in Slovakia. Therefore, we aimed to identify direct and indirect costs of AF in Slovakia, as well as update the data on economic impact of AF-related stroke with the indication of trends in costs and healthcare utilization. Our research effort responds to the recommendation by the 2020 European Society of Cardiology’s Guidelines for the Management of AF [8] and the 2017 HRS/EHRA/ECAS/APHRS/SOLAECE Expert Consensus Statement on Catheter and Surgical Ablation of Atrial Fibrillation [9] calling out to explore the national and regional burden of AF.

## Methods

### Analysis framework and data sources

A population-based study of AF and stroke was conducted from the third-party payer and societal perspective, including healthcare costs, productivity losses, and disability costs. AF and stroke were defined according to the WHO ICD-10 classification [10] i.e., ICD codes of I47–I49 for AF and I60–I69 for stroke. The third-party payer included both private insurance company (Všeobecná zdravotná poisťovňa, DÔVERA zdravotná poisťovňa, and UNION zdravotná poisťovňa) and public payer (Sociálna poisťovňa), that reimburses healthcare providers for services rendered to patients. The choice of codes reflects the real-life practice when AF is reported in the insurance claims also as “supraventricular tachycardia” or “other tachycardia”. Aggregate data on public and private insurance claims was obtained from the Ministry of Health, Institute of Health Analysis, state-owned health insurance companies i.e., the Social Insurance Agency, and the General Health Insurance Company, as well as the private Dôvera health insurance company [11]. Costs and healthcare utilization associated with all reimbursed care for I47–I49 and I60–I69 diagnoses were analysed over the period of five full calendar years (from January 1st 2015 to December 31st 2019). One patient could receive in one year several kinds of care including prescription drugs, diagnostic procedures or could have been hospitalized. All costs were expressed in a respective year. Total prevalence and incidence of both diagnostic groups were obtained from the official health care database managed by the responsible government agency. Aggregate data sets obtained from each source underwent proper verification and cleaning.

### Direct healthcare costs

Direct healthcare costs of AF and stroke included the costs associated with hospitalizations, treatment, and the use of diagnostics. For hospitalizations, all costs associated with inpatient care of longer than 48 h due to the specific diagnosis of AF or stroke were considered. Costs of treatment included all reimbursed pharmacotherapy and/or medical devices reimbursed separately or on top of hospitalization. Diagnostics costs covered all diagnostic procedures that are not covered by hospitalization fee and are done in in-patient or out-patient healthcare settings: including sets of laboratory tests associated with diagnosis, electrocardiography, chest X-ray, echocardiograph, 24 h electrocardiographic monitoring, transesophageal echocardiogram, computed tomography scan, magnetic resonance imaging, arteriography, etc. In accordance with literature data that 30% of strokes occur in people with AF. [1, 12] Therefore we estimated that the same proportion of direct medical costs associated with primary diagnosis of stroke will be associated with AF.

### Indirect costs

Productivity loss and disability costs comprised the indirect costs. The cost associated with productivity losses was estimated as a number of all days spent on paid sick leave from work due to I47–I49 and I60–69 diagnosis provided by the Social Insurance Agency multiplied by the daily rate calculated from the average Slovak salary in industry (Supplementary Table s1).

The disability costs were calculated by multiplying the total number of persons with I47–I49 (AF) and I60–69 (stroke) diagnoses who were granted a formal disability designation by physicians provided by the Social Insurance Agency and a lump sum benefit provided by the Slovak state for the disability (*invalidny dochodok*; Supplementary Table s2)*.* The Social Insurance Agency is responsible for setting up the rules for the benefit associated with disability (https://www.socpoist.sk/invalidny-dochodok/1288s).

Out-of-pocket expenses were not considered, nor caregiver or other indirect costs associated with providing the care to patients by family members or relatives.

### Statistical analysis

We employed a comprehensive approach to analyse the aggregated data on treatments and patient demographics, as well as information on sick leaves and disability pensions. The statistical analysis was conducted in several stages. Initially, we performed basic calculations to disaggregate the data by year and by diagnosis. This step allowed us to assess temporal trends in treatment efficacy, patient outcomes, and the economic impact of sick leaves and disability pensions on the healthcare system and the broader society. For each year and diagnosis, we calculated cost categories, the length of sick leaves, and the allocation of disability pensions.

Subsequent analyses included comparative assessments across different years and diagnoses. We utilized standard statistical techniques to determine the trends and differences. This approach enabled us to draw meaningful conclusions about the dynamics of disease cost and its socioeconomic implications over the study period. The statistical analysis was performed to ensure transparency and reproducibility with the same datasets. All calculations were performed using MS Excel adhering to best practices in data processing and analysis.

## Results

### Population

**Fig. 1:**
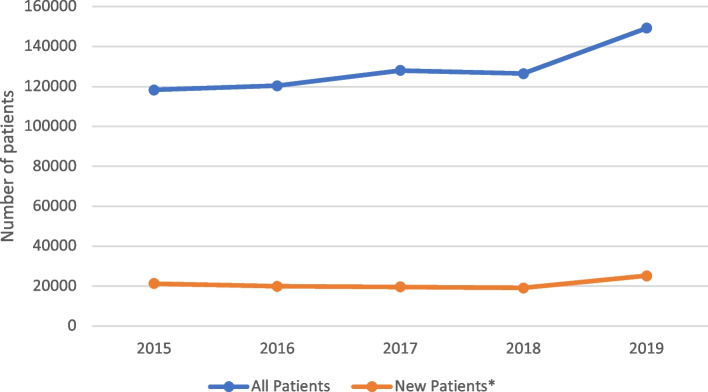
Total prevalence and incidence of AF in Slovakia (ICD-10: I47–I49; 2015–2019). *New patients are defined as those that received any kind of reimbursed care associated with AF diagnosis first time in a respective year Source: Národné centrum zdravotníckych informácií—National Health Information Center (http://www.nczisk.sk/Statisticke_vystupy/Tematicke_statisticke_vystupy/Kardiologia/Pages/default.aspx). AF, atrial fibrillation

**Fig. 2:**
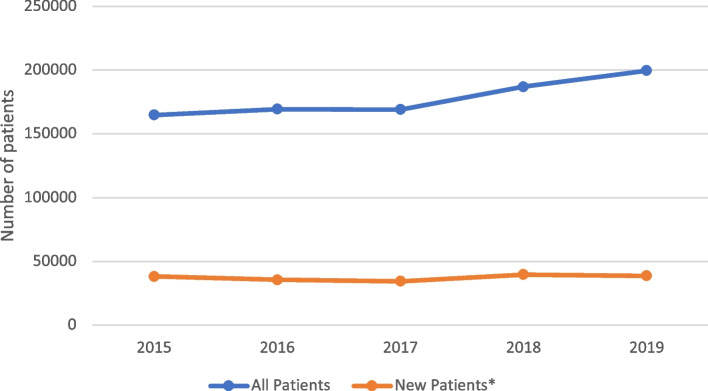
Total prevalence and incidence of stroke (ICD-10: I60–I69; 2015–2019 year). *New patients are defined as those that received any kind of reimbursed care associated with stroke diagnosis first time in a respective year. Source: Národné centrum zdravotníckych informácií—National Health Information Center (http://www.nczisk.sk/Statisticke_vystupy/Tematicke_statisticke_vystupy/Kardiologia/Pages/default.aspx)

According to data obtained from the official health care database, there were 118,341 total cases of AF (I47–I49) in Slovakia in 2015. The prevalence of AF increased by 26% over the 5 years and amounted to a total of 149,198 AF cases in 2019 year. Each year, an average of 20,994 new AF cases that received any kind of reimbursed care associated with the diagnosis was reported (Fig. 1). The total number of reimbursed cases for the stroke patients who were treated with any kind of reimbursed care increased from 164,795 in 2015 year to 199,508 in 2019 year, which represents an average 21% increase rate (Fig. 2).

### Total direct healthcare costs

Over the 5-year period, a total of €269,086,807 direct costs were estimated for AF. Total direct costs increased by €13,881,708 (average of €2,776,342 each year). The impact of hospitalization on total direct costs decreased substantially over the 5-year time horizon, from 41.7% of total direct costs in 2015 year to 27.5% in 2019 year. At the same time, treatment costs became a more prominent total cost driver, increasing from 38% of total direct costs in 2015 year to 53.7% in 2019 year. The share of diagnostic costs in the total cost of care remained at similar level (20.4% in 2015, and 18.9% in 2019) (Table 1).

**Table 1:** Total direct costs of AF in Slovakia (ICD-10: I47–I49; 2015–2019)

Cost category	2015	2016	2017	2018	2019	Total (2015–2019)
Diagnostic costs	€9,882,442	€10,364,691	€10,339,608	€10,774,734	€11,721,146	€53,082,621
Treatment costs	€18,404,988	€21,758,916	€25,057,528	€27,249,406	€33,523,024	€125,993,862
Hospitalization costs	€20,206,667	€19,544,431	€16,550,069	€16,577,522	€17,131,635	€90,010,324
Total costs	**€48,494,097**	**€51,668,038**	**€51,947,205**	**€54,601,662**	**€62,375,805**	**€269,086,807**

*AF* atrial fibrillation

Total direct costs of stroke over the 5-year horizon were estimated at €348,789,666. Total annual direct costs increased from 2015 to 2019 by €28,404,098 (average increase of €5,681,620 each year). The importance of hospitalization as a key direct cost driver increased from 47.2% of total direct costs in 2015 year to 54.3% of total direct costs in 2019 year. The share of diagnostic costs in total direct costs slightly decreased (39% of total direct costs in 2015 year, and 37% of total direct costs in 2019 year). Another noticeable change was observed for treatment costs, which decreased from 13.8% of total direct costs in 2015 year to 8.7% of total direct costs in 2019 year (Table 2). Medical costs of AF-related stroke, estimated as 30% of total annual direct costs of stroke, were €25,734,080 in 2019.

**Table 2:** Total direct costs of stroke in Slovakia (ICD-10: I60–I69; 2015–2019)

Cost category	2015	2016	2017	2018	2019	Total (2015–2019)
Diagnostic costs	€22,387,426	€25,954,487	€24,635,515	€27,578,008	€31,727,844	€132,283,280
Treatment costs	€7,894,952	€7,840,224	€7,634,887	€7,587,150	€7,456,180	€38,413,393
Hospitalization costs	€27,089,791	€27,843,238	€34,789,603	€41,774,120	€46,596,242	€178,092,994
Total costs	€57,372,169	€61,637,948	€67,060,004	€76,939,278	€85,780,267	€348,789,666
Total costs of stroke due to AF	**€17,211,651**	**€18,491,384**	**€20,118,001**	**€23,081,783**	**€25,734,080**	**€104,636,900**

### Total indirect costs

Total 5-year costs associated with absenteeism due to AF were estimated at €20,488,447, whereas the disability costs over the same period amounted to €751,320. Absenteeism costs rose until 2018 year at an average rate of 9% relative to 2015 year (by average of €323,422 each year), and dropped by €1,098,835, which is a 22.8% reduction of 2018 value. Disability costs were subject to more fluctuations over the 5-year period (Table 3). Atrial fibrillation and flutter diagnosis (I48) is the key driver of absenteeism due to AF (I47–I49) accounting for 53.2% of total costs associated with the paid sick leave from work (Supplementary Table s3). Similarly, I48 is responsible for nearly 60% of total disability costs due to AF (Supplementary Table s4).

**Table 3:** Total indirect costs of AF in Slovakia (2015–2019)

Cost category	2015	2016	2017	2018	2019	Total (2015–2019)
Absenteeism	€3,530,551	€3,853,974	€4,554,283	€4,824,237	€3,725,402	€20,488,447
Disability	€155,554	€158,241	€161,298	€135,074	€141,152	€751,320
Total indirect costs	€3,686,105	€4,012,215	€4,715,581	€4,959,311	€3,866,554	€21,239,767

*AF* atrial fibrillation

When it comes to stroke, the total 5-year costs associated with absenteeism due to stroke were estimated at €2,468,881, whereas the disability cost over the same period amounted to €9,689,937. Absenteeism costs rose gradually by 14.2% and averaged €17,673 annually until 2018 (compared to 2015). In 2019 a drop of €221,701 was observed (reduction of 40% compared to preceding year 2018). Similar dynamic characterized the disability costs over the 5-year period (Table 4). Cerebral infarction diagnosis (I63) is the key driver of absenteeism due to stroke (I60–I69) accounting for 53.3% of total costs associated with the paid sick leave from work (Supplementary Table s5). Total disability costs of I63 exceed 43% of total disability costs due to all AF diagnoses (Supplementary Table s6).

**Table 4:** Total indirect costs of stroke (2015–2019)

Cost category	2015	2016	2017	2018	2019	Total (2015–2019)
Absenteeism	€497,884	€524,421	€531,129	€568,574	€346,873	€2,468,881
Disability	€1,817,258	€1,939,127	€1,966,401	€2,002,418	€1,964,732	€9,689,937
Total indirect costs	€2,315,142	€2,463,548	€2,497,530	€2,570,992	€2,311,605	€12,158,818

## Discussion

The results of our study provide a much needed up-to-date information on the direct and indirect costs of AF, as well as the economic impact of AF-related stroke in Slovakia. Based on data from public and private insurance claims in Slovakia, we estimated that the total annual direct medical costs of AF were approximately €62,4 million in 2019, representing 94% of the total costs of AF, while the medical costs of stroke were approximately €85,8 million (97% of the total costs of stroke).

Stroke represents a significant public health and societal burden in the ageing population of the western world. Consistent with clinical experience, stroke was shown to be significantly more severe in patients with AF (0–14 points on the Scandinavian Stroke Scale). Severe stroke occurred in 13.7% of patients with AF compared to 7.9% of patients without AF. As a result, the absolute rates of 30‐day and 1‐year mortality were significantly higher in patients with AF (12.1% and 28.4%, respectively) than in patients without AF (8.7% and 21.8%, respectively) [13]. According to previously published data, the crude incidence of stroke in the adult Slovak population (> 20 years), calculated from the national registry managed by the National Health Information Centre increased continuously, from 138/100 000 persons in 2007 to 211/100 000 in 2018 [14]. The 1-year mortality rate in patients with AF was almost 2 times higher than in patients without AF. For all patients after a first stroke, the 1-year mortality in the year 2017 was 21% (17% for men and 26% for women) [14]. Importantly, 3 out of 4 deaths occurred in the first 3 months after stroke. Recent data from the Danish Stroke Registry showed a comparable 1-year cumulative mortality after first stroke of 28.4% in patients with AF and 21.8% in those without AF [13].

According to data collected in the hospitalization database (report of inpatient hospital admissions), a total of 12,144 patients were hospitalized with a principal diagnosis of AF (ICD-10: I48) in 2014 (the total number of hospitalizations in these individuals was 15,605). From this patient cohort, 963 (7.9%) patients had a stroke within 4 years (2014–2017) after the index hospitalization for AF. This relatively low incidence of stroke in patients previously hospitalized with AF suggests that adequate thromboprophylaxis with anticoagulants is given after discharge from hospital. However, these patients probably represent only a small proportion of AF patients who have a stroke. It is generally accepted that 20–30% of all ischemic strokes are of thromboembolic aetiology, mainly due to AF. Therefore, we must assume that a significant proportion of patients with thromboembolic stroke in Slovakia are patients with subclinical AF, together with patients with known AF who are not on adequate anticoagulation. This population is the main target of efforts to reduce the risk of stroke. To justify such efforts, the cost-effectiveness of available management strategies should be assessed not only in terms of the expected reduction in strokes, but also in terms of the reduction in mortality within 1 year of the event (this is the critical period for the outcome of stroke patients). However, in order to assess the cost-effectiveness of any management strategy for AF, it is of utmost importance to know the true global expenditure of stroke, which includes not only direct healthcare costs but also the necessary social care require by patients and their families after stroke. To our knowledge, the present data are the first attempt to estimate such global expenditure. Of course, the costs of healthcare and social services are highly country-specific and linked to the overall economic performance of the country. Nevertheless, our results may serve as a useful guide for several countries in Central and Eastern Europe with health and social care systems and per capita expenditure comparable to Slovakia’s.

AF represent a significant economic burden, with the management of patients with this condition estimated to account for up to 2.6% of annual healthcare expenditure in European countries [15]. However, the direct and indirect costs of AF management vary widely between countries. Estimates of the total annual cost per patient with AF range from €3209 in France to €7688 in Germany. A systematic review conducted by Wolowacz et al. highlighted the high direct medical costs of AF in Europe, ranging from €450 to €3000 per patient per year, with inpatient care accounting for 50–70% of annual direct costs [16]. Furthermore, a population-based cost-of-illness study from Denmark showed that the costs over a 3-year period for AF patients with stroke were significantly higher than for AF patients without stroke (mean cost per patient over a 3-year period: €30,066 vs. €89,510) [17]. In this case, the mean cost of hospitalization was the main cost driver in the total direct and indirect costs attributable to ischemic stroke and was estimated to be €48,580 (mean total cost per patient with stroke: €59,443).

Assuming that about 30% of strokes are due to AF, we estimated that the annual cost of medical treatment of AF-related strokes in Slovakia could be as high as €25,7 million. In this context, reducing the rate of hospitalization of AF patients, especially from stroke, seems to be the most important factor in controlling the cost of medical treatment. Estimating the expected costs of AF-related strokes also shows a proportion of costs that could be prevented by appropriate anticoagulation therapy. According to published studies, oral anticoagulation reduces the risk of stroke by 64% and mortality by 26% in patients with AF [18, 19]. In the meta-analysis of randomized controlled trials of oral anticoagulation, warfarin reduced the risk of stroke in patients with AF by approximately 60% [19]. In large randomized control trials, anticoagulation with direct oral non-vitamin K antagonists showed improved safety compared with warfarin and an additional 15% risk reduction in the composite endpoint of stroke or systemic embolic events [20]. Given the high cost of treating AF-related stroke, the results of the above clinical trials underscore both the need for screening for subclinical AF in high-risk populations and the subsequent early implementation of effective strategies not only for anticoagulation but also for rhythm control, if appropriate [21].

The economic burden of stroke and AF-associated stroke has been the subject of several European studies [22–24]. In all previous analyses, the authors found a high proportion of direct costs associated with stroke, mainly related to hospitalization. Probably the most comprehensive estimate by Luengo-Fernandez and colleagues for 32 European countries showed that in 2017, the total direct and indirect costs of stroke in these populations were approximately €60 billion, with healthcare costs accounting for 45% of the total (€27 billion) [22]. Importantly, medical costs associated with stroke accounted for about 1.7% of total European health expenditure. For Slovakia, these costs were estimated at €347 million and €177 million, respectively, with medical costs representing 3.1% of total annual health expenditure. In our analysis, the total annual costs were lower, and estimated at €88.1 million. These differences are mainly due to the high degree of variation between the cost estimation methodologies used in both analyses and the different cost categories included in the estimates. The cost estimates by Luengo-Fernandez and colleagues are based rather on global assumptions (such as combined data form several Central and Eastern European countries from the SHARE study [Survey of Health, Ageing and Retirement in Europe] or analysis by the Organisation for Economic Cooperation and Development – OECD) rather than on real data. The much lower reimbursement for healthcare services combined with lower hourly rates for health care providers in Slovakia may explain these differences. In contrast to these estimates, our assessment of the costs of diagnosis, treatment, and hospitalization of stroke patients is based on aggregated data from public and private insurance claims. There is also a significant difference in the assessment of indirect costs: our study did not include the costs associated with mortality and informal care. In the analysis by Luengo-Fernandez et al., these costs represent a significant proportion of indirect costs (€54 and €83 million, respectively, out of €170 million in indirect costs in 2017). Nevertheless, both analyses demonstrate a substantial health and societal burden of stroke in Slovakia. Furthermore, our data suggest a possible association between low reimbursement costs and excessive 1-year mortality of stroke patients. Unfortunately, the recent further reduction in per capita healthcare spending may further worsen the outcome of these patients.

In our study, the observed proportion of patients hospitalized for stroke (ICD I60–I69) with atrial fibrillation/flutter (ICD I47–I49) diagnosed in the 5 years prior to stroke increased from 22.7% to 36.1% in 2015–2019; data not shown). This is in line with generally accepted epidemiological data [1, 12]. However, such an increase in the Slovakian population could be explained by an increasing number of patients being diagnosed with AF but not receiving appropriate anticoagulation therapy. This gap between diagnosis and necessary therapeutic intervention underlines the need for better implementation of evidence-based guidelines in clinical practice.

Our study has several limitations. First, some diagnoses may be missing from the medical records. As a result, the rates of AF and stroke may be underestimated, leading to an underestimation of the total costs of both conditions, although to minimize this risk we combined data from both public and private insurance claims in Slovakia. The second limitation is the potential underestimation of disability costs due to the inclusion of only formal/official state-granted disability data, which does not cover informal expenditures and costs of sequelae. The analysis by Luengo-Fernandez and colleagues shows that these costs could be an important part of the total burden of disease for stroke and AF. On the other hand, the choice of ICD codes analysed may have contributed to the inclusion of patients with arrhythmias other than AF or flutter. However, in the light of similar analyses from comparable countries, we believe that our assessment is rather conservative and that the total economic burden of AF and AF-related stroke in Slovakia may be higher. Finally, our results should be interpreted for each individual condition, and not cumulatively (considering data from AF and AF-related stroke together). We did not directly calculate the costs associated with AF and stroke, as this could introduce bias due to the need for assumptions and the complexity of the calculations.

## Conclusions

In conclusion, in our study we evaluated the economic burden of AF and AF-related stroke in the Slovak population. The annual direct costs of treatment of patients with AF in Slovakia in 2019 were €62.4 million, and for patients with stroke €85.8 million. Our estimates show that the average annual increase in direct costs for AF was 7%, while for stroke it was 11%. The main cost driver for stroke was hospitalisation, which accounted for 53% of total costs. The high economic burden of both conditions highlights the unmet need to improve the diagnosis of subclinical AF and its early treatment with anticoagulation and, if appropriate, rhythm control.

### Supplementary Information

Supplementary Material 1.

## Data Availability

The datasets used and analysed during the current study are available and can be accessed from the corresponding author on reasonable request.

## Abbreviation

AF: Atrial fibrillation
