# Supplementary material for: Epidemiology and comprehensive economic impact of atrial fibrillation and associated stroke in Slovakia
Source: BMC Health Serv Res. 2024 May 17;24:637. doi: 10.1186/s12913-024-11100-1 (PMC11100086; doi:10.1186/s12913-024-11100-1)
Supplement: Supplementary file 1 — Supplementary Material 1. [file 12913_2024_11100_MOESM1_ESM.docx]

Table s1 Average salary in industry in Slovakia (2015–2019)

| **Year** | **Value/ Unit value** |
| --- | --- |
| 2015 | €883 |
| 2016 | €912 |
| 2017 | €954 |
| 2018 | €1,013 |
| 2019 | €1,092 |

Source: https://www.finance.sk/mzda/priemerna-mzda/

Table s2 Disability lump sum rates in Slovakia (2015–2019)

| **Year** | **Formally confirmed disability level** | |
| --- | --- | --- |
|  | **< 70%** | **≥ 70%** |
| 2015 | €197.51 | €352.49 |
| 2016 | €197.04 | €353.18 |
| 2017 | €200.05 | €359.88 |
| 2018 | €203.69 | €368.03 |
| 2019 | €209.85 | €379.95 |

Source: https://www.socpoist.sk/priemerna-vyska-vyplacanych-dochodkov--v-mesiacoch-/1600s, ku 31.12.2019

Table s3 Total costs associated with the paid sick leave from work due to AF in Slovakia (2015–2019)

| **ICD-10 code** | **2015** | **2016** | **2017** | **2018** | **2019** |
| --- | --- | --- | --- | --- | --- |
| **I47** | €830,122 | €858,681 | €934,013 | €983,356 | €851,570 |
| **I48** | €1,819,974 | €2,038,595 | €2,610,972 | €2,734,100 | €1,981,646 |
| **I49** | €880,456 | €956,698 | €1,009,298 | €1,106,780 | €892,186 |
| **Total costs** | €3,530,552 | €3,853,974 | €4,554,283 | €4,824,237 | €3,725,402 |

AF, atrial fibrillation

Table s4 Total number of patients with disability due to AF (2015–2019)

| **ICD-10 code** | **2015** | **2016** | **2017** | **2018** | **2019** | **Total** | **% share** |
| --- | --- | --- | --- | --- | --- | --- | --- |
| **I47** | 5 | 6 | 2 | 6 | 11 | 30 | 10.87% |
| **I48** | 33 | 30 | 42 | 31 | 29 | 165 | 59.78% |
| **I49** | 19 | 23 | 16 | 11 | 12 | 81 | 29.35% |
| **Total** | 57 | 59 | 60 | 48 | 52 | 276 | 100.00% |

Source: Social Insurance Agency, 2020

AF, atrial fibrillation

Table s5 Total costs associated with the paid sick leave from work due to stroke (2015–2019)

| **ICD-10 code** | **2015** | **2016** | **2017** | **2018** | **2019** |
| --- | --- | --- | --- | --- | --- |
| **I60** | €453,787 | €469,500 | €477,060 | €511,987 | €344,768 |
| **I61** | €563,273 | €666,541 | €875,479 | €925,867 | €534,038 |
| **I62** | €85,946 | €70,200 | €83,140 | €103,545 | €73,598 |
| **I63** | €3,907,153 | €4,162,504 | €4,699,976 | €5,422,939 | €3,613,662 |
| **I64** | €1,767,879 | €1,940,417 | €2,030,630 | €2,124,291 | €1,314,314 |
| **I65** | €98,976 | €108,354 | €185,515 | €206,263 | €149,120 |
| **I66** | €41,950 | €43,169 | €38,004 | €32,146 | €26,277 |
| **I67** | €561,137 | €678,429 | €368,246 | €705,046 | €442,066 |
| **I68** | €56,118 | €55,279 | €57,052 | €54,954 | €40,280 |
| **I69** | €341,374 | €357,799 | €245,476 | €227,199 | €238,262 |
| **Total costs** | €7,877,593 | €8,552,192 | €9,060,578 | €10,314, 237 | €6,776,385 |

Table s6 Total number of patients with disability due to stroke (2015–2019)

| **ICD-10 code** | **2015** | **2016** | **2017** | **2018** | **2019** | **Total** | **% share** |
| --- | --- | --- | --- | --- | --- | --- | --- |
| **I60** | 24 | 35 | 31 | 20 | 23 | 133 | 4.45% |
| **I61** | 59 | 56 | 51 | 72 | 54 | 292 | 9.77% |
| **I62** | 2 | 3 | 1 | 3 | 6 | 15 | 0.50% |
| **I63** | 243 | 268 | 251 | 252 | 274 | 1,288 | 43.11% |
| **I64** | 52 | 58 | 42 | 24 | 28 | 204 | 6.83% |
| **I65** | 6 | 7 | 13 | 11 | 10 | 47 | 1.57% |
| **I66** | 3 | 2 | 1 | 2 | 1 | 9 | 0.30% |
| **I67** | 37 | 26 | 51 | 42 | 34 | 190 | 6.35% |
| **I68** | 5 | 1 | 0 | 0 | 0 | 6 | 0.20% |
| **I69** | 138 | 167 | 172 | 177 | 150 | 804 | 26.91% |
| **Total** | 569 | 623 | 613 | 603 | 580 | 2988 | 100.00% |

Source: Social Insurance Agency, 2020
